# Supplementary material for: Sexual antagonism evolves when autosomes influence offspring sex ratio
Source: bioRxiv. 2023 Jun 15:2023.06.14.544982. Preprint. [Version 1] doi: 10.1101/2023.06.14.544982 (PMC10312671; doi:10.1101/2023.06.14.544982)
Supplement: 2 [file NIHPP2023.06.14.544982v1-supplement-2.pdf]

bioRxiv preprint doi: https://doi.org/10.1101/2023.06.14.544982; this version posted June 15, 2023. The copyright holder for this preprint (which was not certified by peer review) is the author/funder, who has granted bioRxiv a license to display the preprint in perpetuity. It is made available under aCC-BY 4.0 International license.

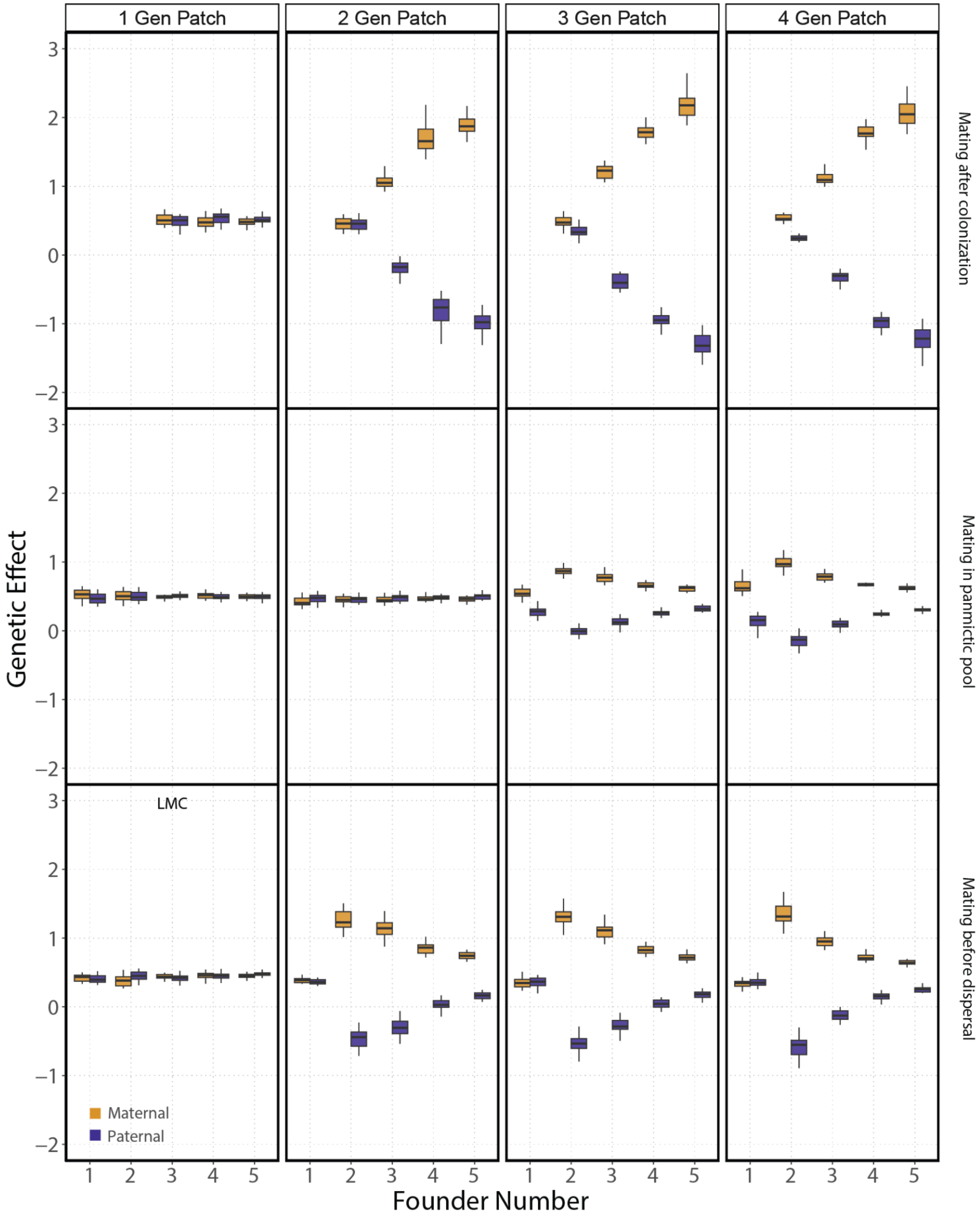

521 **Supplemental Figure 1.** Comparing models of metapopulation dispersal and generations within a patch
522 on evolved genetic effects for simulations with only maternal or paternal inheritance modes with discrete
523 generations. Box plots for the genetic effects in generations 2588-2592 depending on the number of
524 generations in a stack and the model (each condition was run 20 times; brood size = 5). A minimum of
525 two founders is required for the mating after colonization model, additionally, all populations in the two-
526 founder single-generation patch experienced extinction.

bioRxiv preprint doi: https://doi.org/10.1101/2023.06.14.544982; this version posted June 15, 2023. The copyright holder for this preprint (which was not certified by peer review) is the author/funder, who has granted bioRxiv a license to display the preprint in perpetuity. It is made available under aCC-BY 4.0 International license.

Supplemental Text

Here we show single-locus recursion equations for a single patch, adapted from Wilson and Colwell's (1981) haplodiploid model to the autosomal parental-effect diploid case with arbitrary dominance. These equations track the number of individuals of each sex and genotype in a growing patch. Terms that differ between the maternal- and paternal-effect models are highlighted in red.

N_f	Number of females
N_m	Number of males
N	Number of individuals $(N_f + N_m)$
$p_{f11}, p_{f12}, p_{f22}$	Genotype frequencies among females (sum to 1)
$p_{m11}, p_{m12}, p_{m22}$	Genotype frequencies among males (sum to 1)
p_{f1}, p_{f2}	Allele frequencies among females (sum to 1)
p_{m1}, p_{m2}	Allele frequencies among males (sum to 1)
E	Number of offspring per female
r	Sex ratio (N_m/N) in broods of $_{11}$ homozygotes of the ratio-controlling parent
s	Shift of sex ratio in broods of $_{22}$ homozygotes of the ratio-controlling parent
h	Dominance coefficient (i.e., ratios of $_{11}$, $_{12}$, and $_{22}$ are $r, r + hs, r + s$)

Maternal-effect Recursion Equations

$$(N_f p_{f11})'_{mat} = N_f E(p_{f1} p_{m1}(1-r) - \frac{p_{f12} p_{m1}}{2} hs)$$

$$(N_f p_{f12})'_{mat} = N_f E\left((p_{f1} p_{m2} + p_{f2} p_{m1})(1-r) - (\frac{p_{f12}}{2} h + p_{f22} p_{m1})s\right)$$

$$(N_f p_{f22})'_{mat} = N_f E(p_{f2} p_{m2}(1-r) - p_{m2}(\frac{p_{f12}}{2} h + p_{f22})s)$$

$$(N_m p_{m11})'_{mat} = N_f E(p_{f1} p_{m1} r + \frac{p_{f12} p_{m1}}{2} hs)$$

$$(N_m p_{m12})'_{mat} = N_f E\left((p_{f1} p_{m2} + p_{f2} p_{m1})r + (\frac{p_{f12}}{2} h + p_{f22} p_{m1})s\right)$$

$$(N_m p_{m22})'_{mat} = N_f E(p_{f2} p_{m2} r + p_{m2}(\frac{p_{f12}}{2} h + p_{f22})s)$$

Paternal-effect Recursion Equations

$$(N_f p_{f11})'_{pat} = N_f E(p_{f1} p_{m1}(1-r) - \frac{p_{m12} p_{f1}}{2} hs)$$

$$(N_f p_{f12})'_{pat} = N_f E\left((p_{f1} p_{m2} + p_{f2} p_{m1})(1-r) - (\frac{p_{m12}}{2} h + p_{m22} p_{f1})s\right)$$

$$(N_f p_{f22})'_{pat} = N_f E(p_{f2} p_{m2}(1-r) - p_{f2}(\frac{p_{m12}}{2} h + p_{m22})s)$$

$$(N_m p_{m11})'_{pat} = N_f E(p_{f1} p_{m1} r + \frac{p_{m12} p_{f1}}{2} hs)$$

$$(N_m p_{m12})'_{pat} = N_f E\left((p_{f1} p_{m2} + p_{f2} p_{m1})r + (\frac{p_{m12}}{2} h + p_{m22} p_{f1})s\right)$$

$$(N_m p_{m22})'_{pat} = N_f E(p_{f2} p_{m2} r + p_{f2}(\frac{p_{m12}}{2} h + p_{m22})s)$$

bioRxiv preprint doi: https://doi.org/10.1101/2023.06.14.544982; this version posted June 15, 2023. The copyright holder for this preprint (which was not certified by peer review) is the author/funder, who has granted bioRxiv a license to display the preprint in perpetuity. It is made available under aCC-BY 4.0 International license.

The within-patch differences between maternal and paternal control of the sex ratio can be illustrated with a simple numerical example involving a patch with three male and three female founders. In each patch, an allele that shifts offspring sex ratio from 0.5 to 0.35 to 0.2 with each additional allele in the parental genotype starts at frequency 0.5 in the founding generation. Each female has 10 offspring.

This example uses the following model parameters:
$N_f = 3$, $N_m = 3$, $p_{f11} = 0.25$, $p_{f12} = 0.5$, $p_{f22} = 0.25$, $p_{m11} = 0.25$, $p_{m12} = 0.5$, $p_{m22} = 0.25$, $r = 0.5$, $s = -0.3$, $h = 0.5$, $E = 10$.

Paternal Control

Generation	Individual Count	Sex Ratio	Allele Frequency
1	6	0.5	0.500
2	30	0.350	0.500
3	195	0.366	0.488
4	1236.2	0.369	0.477
5	7803.6	0.371	0.467

Maternal Control

Generation	Individual Count	Sex Ratio	Allele Frequency
1	6	0.5	0.500
2	30	0.350	0.500
3	195	0.341	0.488
4	1284.4	0.345	0.474
5	8409.8	0.349	0.462
